# Fibroblast Growth Factor 21 Is Not Required for the Reductions in Circulating Insulin-Like Growth Factor-1 or Global Cell Proliferation Rates in Response to Moderate Calorie Restriction in Adult Mice

**DOI:** 10.1371/journal.pone.0111418

**Published:** 2014-11-04

**Authors:** Airlia C. S. Thompson, Matthew D. Bruss, Nitish Nag, Alexei Kharitonenkov, Andrew C. Adams, Marc K. Hellerstein

**Affiliations:** 1 Department of Nutritional Science and Toxicology, University of California, Berkeley, California, United States of America; 2 Lilly Research Laboratories, Lilly Corporate Center, Indianapolis, Indiana, United States of America; 3 KineMed, Inc., Emeryville, California, United States of America; University of Navarra School of Medicine and Center for Applied Medical Research (CIMA), Spain

## Abstract

Calorie restriction (CR) delays aging and extends lifespan in numerous organisms, including mice. Down-regulation of the somatotropic axis, including a reduction in insulin-like growth factor-1 (IGF-1), likely plays an important role in CR-induced lifespan extension, possibly by reducing cell proliferation rates, thereby delaying replicative senescence and inhibiting tumor promotion. Accordingly, elucidating the mechanism(s) by which IGF-1 is reduced in response to CR holds therapeutic potential in the fight against age-related diseases. Up-regulation of fibroblast growth factor 21 (FGF21) is one possible mechanism given that FGF21 expression is induced in response to nutritional deprivation and has been implicated as a negative regulator of IGF-1 expression. Here we investigated alterations in hepatic growth hormone (GH)-mediated IGF-1 production and signaling as well as the role of FGF21 in the regulation of IGF-1 levels and cell proliferation rates in response to moderate CR in adult mice. We found that in response to moderate CR, circulating GH and hepatic janus kinase 2 (JAK2) phosphorylation levels are unchanged but that hepatic signal transducer and activator of transcription 5 (STAT5) phosphorylation levels are reduced, identifying STAT5 phosphorylation as a potential key site of CR action within the somatotropic axis. Circadian measurements revealed that the relative level of FGF21 expression is both higher and lower in CR vs. ad libitum (AL)-fed mice, depending on the time of measurement. Employing FGF21-knockout mice, we determined that FGF21 is not required for the reduction in IGF-1 levels or cell proliferation rates in response to moderate CR. However, compared to AL-fed WT mice, AL-fed FGF21-knockout mice exhibited higher basal rates of cell proliferation, suggesting anti-mitotic effects of FGF21. This work provides insights into both GH-mediated IGF-1 production in the context of CR and the complex network that regulates FGF21 and IGF-1 expression and cell proliferation rates in response to nutritional status.

## Introduction

Calorie restriction (CR), reduced caloric intake without malnutrition, increases maximum lifespan and delays the onset of many age-related diseases in organisms ranging from worms to rodents, and possibly non-human primates [1], [2]. Decreased signaling through the somatotropic axis is one mechanism that has been suggested to mediate these effects of CR [3], perhaps through a reduction in cell proliferation, which is predicted to contribute to lifespan extension by delaying cellular replicative senescence and inhibiting the promotional phase of carcinogenesis [4]. Several lines of evidence contribute to a strong case for this hypothesis. First, CR in mice leads to a reduction in circulating levels of insulin-like growth factor-1 (IGF-1) in association with reduced rates of proliferation in a number of cell types [5]–[9]. Second, repletion of circulating IGF-1 levels in CR rodents attenuates the CR-induced reduction in cell proliferation [10]–[12]. Last, disruption of IGF-1 signaling in several mouse models mimics many of the effects of CR including, increased maximum lifespan, reduced tumor progression, delayed cellular replicative senescence and reduced rates of cell proliferation [4], [13]–[18]. Thus, identifying mechanisms that regulate IGF-1 signaling and cell proliferation in response to CR in mice could provide insight into the biology of aging and offer therapeutic targets for treating age-related diseases.

Regulation of hepatic IGF-1 production, the predominant source of circulating levels [19], is well understood under *ad libitum* (AL) feeding conditions. Circulating growth hormone (GH) binds to the GH receptor (GHR) stimulating its association with the intracellular kinase, janus kinase 2 (JAK2), which subsequently autophosphorylates and phosphorylates the GHR creating a binding site for signal transducer and activator of transcription 5 (STAT5). Once bound to the GHR, STAT5 is phosphorylated by JAK2, which stimulates its dimerization and translocation to the nucleus where STAT5 activates the transcription of IGF-1 [20], [21]. Based on this mechanism, it might be predicted that reduced circulating IGF-1 in CR mice could simply be due to reduced circulating GH. However, while mice on a severe CR diet (50–60% CR) have reduced circulating IGF-1, they actually have increased levels of circulating GH compared to AL controls [22], [23]. High circulating GH levels coincident with low IGF-1 levels have also been observed in humans under conditions of energy restriction [24]. In addition, pulsatile GH secretion appears to be increased in older CR rats, compared to age-matched AL controls [3], [25]. Together these observations suggest that CR regulates IGF-1 expression downstream of GH. The mechanism(s) of CR-induced regulation of IGF-1, however, remain unknown.

Recently, fibroblast growth factor 21 (FGF21) [26], a novel endocrine-like member of the FGF superfamily highly expressed in the liver, has been implicated as a negative regulator of IGF-1 expression and has also been reported to extend lifespan in mice when over-expressed [22], [27]–[30]. Indeed, FGF21 transgenic mice are smaller, exhibit reduced hepatic GH sensitivity downstream of JAK2, have reduced circulating IGF-1 levels and exhibit a 36% increase in median lifespan relative to WT controls [28], [30]. In addition, treatment of WT mice with recombinant human FGF21 reduces circulating IGF-1 levels, while treatment of chondrocytes with FGF21 attenuates GH-induced IGF-1 mRNA expression [28], [29]. Furthermore, long-term CR (74wks) in mice and severe CR (50%CR) in young developing mice increases FGF21 expression relative to AL controls [22], [31]. Importantly, in studies using whole-body FGF21-knockout (FGF21-KO) mice, FGF21 is required to permit the full undernutrition-related reduction in both hepatic mRNA and circulating IGF-1 levels [22].

Interestingly, earlier work demonstrated that circulating FGF21 and hepatic FGF21 mRNA levels are rapidly increased in response to fasting in a peroxisome proliferator-activated receptor alpha (PPARα)-dependent manner [32]–[35]. Given the intermittent nature of feeding in CR mice (∼1 h of gorging upon food provision followed by ∼23 h of fasting) [36], FGF21 expression may be increased in mice even on a moderate CR regimen and this hormonal signaling could lead to the reductions in circulating IGF-1 levels and cell proliferation rates that are observed in response to similar moderate CR regimens [8]. However, the effect of moderate CR in adult mice on FGF21 expression and the role of FGF21 in mediating the IGF-1 and cell proliferation responses to this CR regimen have not been previously explored.

Accordingly, the goal of the present work was to determine the effect of moderate CR (25% CR) on GH secretory dynamics, hepatic GH signaling and FGF21 expression in adult C57BL/6 male mice. In addition, we sought to determine whether FGF21 is necessary for the reductions in circulating IGF-1 levels and cell proliferation rates in response to this CR regimen.

## Materials and Methods

### Animals

12- to 16-week-old male C57BL/6 mice were used in the initial studies (Jackson Laboratory, Bar Harbor, ME). For the FGF21-KO study, 12- to 17-week-old male control C57BL/6NTac (WT) mice and 12- to 15-week-old male FGF21-KO mice, generated as previously described [37], were used (Taconic Farms Inc., Hudson, NY). All mice were housed individually, provided free access to water and maintained under temperature- and light-controlled conditions (12∶12-h light-dark cycle, lights on at 0700 h and off at 1900 h).

### Ethics statement

All protocols and procedures were conducted in strict accordance with accepted standards of humane animal care and approved by the University of California Berkeley Animal Use Committee (Animal Use Protocol Number: R094–0313).

### Diets and feeding regimens

All mice in the AL groups were provided free access to semi-purified AIN-93 M diet (Research Diets, New Brunswick, NJ). All mice in the CR groups were provided semi-purified dustless precision AIN-93M diet pellets (Bio-Serv, Frenchtown, NJ). The average daily food intake of the AL groups was determined every 3 to 4 days and the food provided to the corresponding CR groups was adjusted accordingly. All mice in the CR groups were fed daily at 1600 h.

For the initial studies, following one week of acclimation to the AIN-93 M diet, mice were randomly assigned to one of two dietary groups: AL or CR. Mice in the CR groups were provided with 75% of the average daily food intake of their AL group counterparts (from the previous 3 to 4 days) and, therefore, were 25% calorie restricted. Mice were kept on their feeding regimens for a total of 4 or 6.5 weeks. The body weight of each mouse was determined at least once per week. Mice in these initial studies were euthanized at 2100 h or 2200 h.

For the FGF21-KO study, following one week of acclimation to the AIN-93 M diet, WT and KO mice were randomly assigned to one of two dietary groups: AL or CR. The groups included: WT/AL, WT/CR, FGF21-KO/AL and FGF21-KO/CR. Mice in the CR groups were initially acclimated to the CR regimen over the course of 5 days (10% CR for 2 days, 15% CR for 1 day, 20% CR for 2 days and 25% CR thereon after). All mice were kept on their feeding regimens for a total of 6 weeks. The body weight of each mouse was determined at least twice per week. Half of the mice in each of the four groups were euthanized at 1500 h and the other half at 1900 h.

### Heavy water (^2^H_2_O) labeling

In order to measure global rates of DNA synthesis as a marker of cell proliferation rates, mice in the FGF21-KO study were labeled with an intraperitoneal injection of 100% heavy water (0.35 ml/10g body weight) 3 weeks prior to the end of the study and were then provided free access to 8% heavy water as drinking water for the remainder of the study, as previously described [38].

### Blood collection, tissue collection and cell isolation

#### Blood

Upon completion of each study, mice were anesthetized under 3% isoflurane and blood was collected via cardiac puncture, followed by cervical dislocation, tissue collection and in some cases cell isolation.

#### Liver

Upon dissection, the liver was cut into several small pieces (∼15–50 mg), which were flash frozen in liquid nitrogen. For DNA synthesis measurements, liver samples were homogenized and total DNA from all liver cells was isolated.

#### Keratinocytes, splenic T-cells and bone marrow cells

Keratinocytes, splenic T-cells and bone marrow cells were isolated prior to DNA isolation as previously described [8].

### DNA isolation

DNA was extracted using DNeasy kits (Qiagen, Valencia, CA, USA). Briefly, isolated keratinocytes, liver, T-cells and bone marrow cells were digested overnight at 37C in proteinase K solution followed by DNA isolation according to the manufacturer's instructions and elution into 200 ul water.

### DNA synthesis measurement

Determination of ^2^H incorporation into purine deoxyribose (dR) of DNA was performed as previously described [8]. The enrichment of ^2^H in purine dR was determined by measuring the fractional molar isotope abundances at *m*/*z* 435 (M0 mass isotopomer) and 436 (M1 mass isotopomer) of the pentafluorobenzyl triacetyl derivative of purine dR. Excess fractional M1 enrichment (EM1) was calculated as:
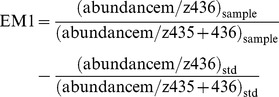
where sample and standard (std) refer to the analyzed sample and an unenriched pentafluorobenzyl triacetyl purine dR derivative standard, respectively. The fractional synthetic rate (*f,* % newly divided cells) of keratinocytes, liver and T-cells was calculated by a comparison to bone marrow cells in the same animal, which represent an essentially fully turned over population of cells.



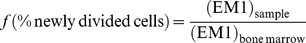



The replacement rate (*k*, % newly divided cells per day) was calculated as:
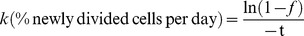
where t is the number of days of exposure to heavy water.

### Blood collection for GH measurement

∼25 ul of blood was collected every 45 min between 1530 h and 2045 h via the tail vein. CR mice were fed at their regular feeding time (1600 h) on the day of blood collection for GH measurement.

### ELISAs

Following centrifugation of blood, plasma was collected and stored at −20C. GH levels were determined in singlet, due to the need to minimize the total volume of blood drawn over the sampling period, with an inter-assay coefficient of variation (CV) of 8.7–11.1% (Millipore, Billerica, MA). FGF21 levels were determined in duplicate with an intra-assay CV and an inter-assay CV of 4.2–16.6% and 9.7–18.7%, respectively (Millipore, Billerica, MA). IGF-1 levels were determined in duplicate (with the exception of one assay done in singlet) with an intra-assay CV and an inter-assay CV of 2.3–6.0% and 7.8%, respectively (R&D Systems, Minneapolis, MN). Insulin-like growth factor binding protein-1 (IGFBP-1) levels were determined in duplicate with an intra-assay CV of 5.34% (Insight Genomics, Falls Church, VA).

### Western blot analysis

Frozen livers were homogenized in 450 ul lysis buffer (10 mM Tris-base, 150 mM NaCl, pH ∼7.5, 1% NP-40, 0.1% SDS, 0.5% sodium deoxycholate, 1 mM dithiothreitol, 1mM phenylmethylsulfonyl fluoride, 7.5 ug/mL leupeptin, 1.0 ug/mL pepstatin, 2.0 ug/mL aprotinin and 1 Phosphatase Inhibitor Cocktail tablet (Roche Applied Science, Indianapolis, IN) per 10 mL buffer) using a stainless steel bead and a TissueLyserII (Retsch, Newtown, PA) set at 30 hz for 1 min. Protein homogenates were sonicated in a sonication bath for 1vmin and then centrifuged at 10,000 rcf at 4C for 10 min followed by supernatant collection. Protein concentrations were determined by bicinchoninic acid assay (Pierce, Rockford, IL) and proteins were fractionated via SDS-PAGE. Proteins were transferred to nitrocellulose membranes (Invitrogen, Grand Island, NY) and probed with the following primary antibodies: total-JAK2 rabbit monoclonal antibody raised against a synthetic peptide corresponding to residues surrounding Pro841 of JAK2 (Cell Signaling Technology, Danvers, MA, catalog number: 3230, used at 1∶250 dilution); phospho-JAK2 (Try 1007/1008) rabbit monoclonal antibody raised against a synthetic phosphopeptide corresponding to a region surrounding Tyr1007/1008 of human JAK2 (Cell Signaling Technology, Danvers, MA, catalog number: 3776, used at 1∶250 dilution); total-STAT5 rabbit polyclonal antibody raised against a synthetic peptide corresponding to an amino-terminal region within STAT5 (Cell Signaling Technology, Danvers, MA, catalog number: 9363, used at 1∶500 dilution); phospho-STAT5 (Tyr694) rabbit monoclonal antibody raised against a synthetic peptide corresponding to residues surrounding Tyr694 of human STAT5a protein (Cell Signaling Technology, Danvers, MA, catalog number: 4322, used at 1∶500 dilution); beta-actin mouse monoclonal antibody raised against gizzard actin of avian origin (Santa Cruz Biotechnology, Inc., Santa Cruz, CA, catalog number: 47778, used at 1∶200 dilution). Blots were incubated with IRDye700DX- or IRDye800CW-conjugated secondary antibodies (Rockland Immunochemicals Inc., Gilbertsville, PA). Protein bands were imaged and densitometry measurements were made using the Odyssey Infrared Imaging System (LI-COR, Lincoln, NE).

### Gene expression

RNA was isolated from snap-frozen liver tissue using RNeasy kits (Qiagen, Valencia, CA) and reverse transcribed with M-MulV reverse transcriptase (New England Biolabs, Ipswich, MA). Next, diluted cDNAs were run on an ABI 7500 Fast Real-Time PCR or ABI PRISM 7900HT Sequence Detection System using TaqMan gene expression master mix and TaqMan probes for FGF21 (Mm00840165_g1), IGF-1 (Mm00439561_m1), IGFBP-1 (Mm00515154_m1) or 18S rRNA (Mm03928990_g1) according to the manufacturer's instructions (Applied Biosystems, Carlsbad, CA). Expression of FGF21, IGF-1 and IGFBP-1 was normalized to 18S rRNA expression.

### Statistical analyses

All results are presented as mean ± SEM. For initial studies, differences between AL and CR groups were analyzed by Student's unpaired two-tailed *t*-tests. For comparisons between WT and FGF21-KO mice fed AL or CR, to analyze differences in body weight and food intake over time, single time point comparisons were made using a one-way ANOVA followed by a Tukey *post hoc* test. Otherwise, differences between groups were analyzed by a two-way ANOVA followed by a Bonferroni *post hoc* test. When two time points were analyzed, differences between WT and FGF21-KO mice fed AL or CR were analyzed separately at each time point by a two-way ANOVA followed by a Bonferroni *post hoc* test. Data were analyzed using Prism Graphpad software (version 5.0a).

## Results

### GH secretory dynamics and hepatic GH signaling in CR mice

Initially we set out to map where within the canonical GH-mediated IGF-1 production pathway signaling diverges in CR mice and thus, may account for reported lower IGF-1 levels in CR mice. To confirm previous reports on the GH status of CR rodents and to extend those findings to short-term moderate CR in adult mice, we placed 12- to 16-week-old mice on either an AL or 25% CR diet for a total of 4 or 6.5 weeks.

**Figure 1:**
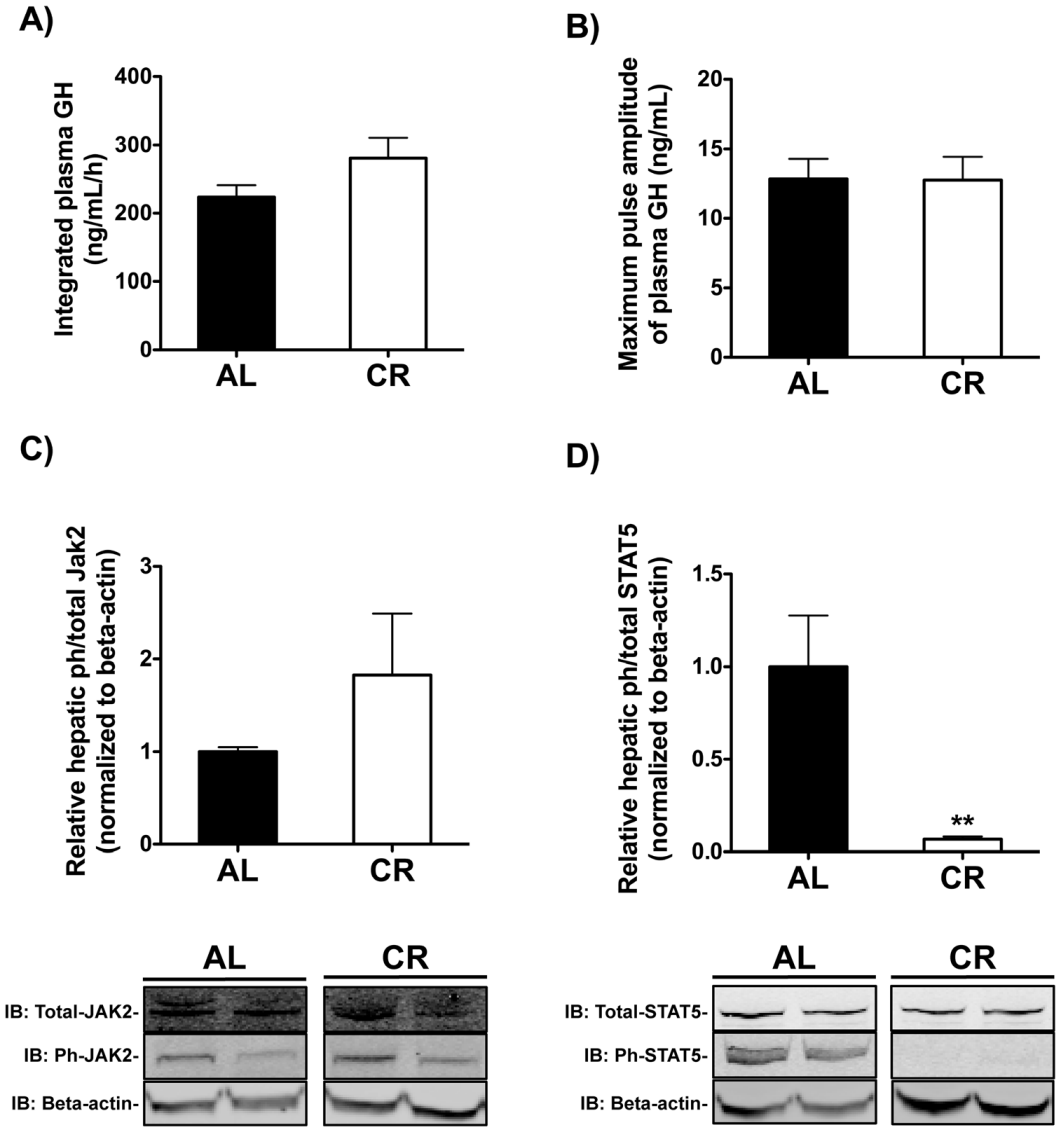
GH secretory dynamics and hepatic GH signaling in CR mice. Plasma GH levels described as A) the integrated hourly concentration or B) the maximum pulse amplitude of GH over the 5.25 h sampling period in AL and CR mice (n = 8 per diet). Densitometric analysis and western blot images of hepatic protein levels of phosphorylated to total C) JAK2 and D) STAT5 in AL and CR mice at 2100 h (n = 5–7 per diet. Data normalized to AL). Student's unpaired two-tailed *t*-tests were used for all between-group analyses (** p<0.0071).

We measured circulating GH levels eight times over a 5.25 h sampling period in mice fed AL or 25% CR for 6.5 weeks. We found that there were no significant differences in circulating GH levels, described as either the integrated concentration or the maximum pulse amplitude of GH, over the 5.25 h sampling period in CR compared to AL mice (**Figure 1A, B** **and Figures S1 and S2 in File S1**). Therefore, a simple reduction in circulating GH levels cannot explain the reduction in IGF-1 levels in response to moderate CR in mice.

To determine whether hepatic GHR signaling is blunted in CR mice on a moderate CR diet, we measured JAK2 and STAT5 phosphorylation as markers of the GHR signaling pathway in this tissue in mice fed AL or 25% CR for 4 weeks. We found that there was no significant difference in hepatic JAK2 phosphorylation in CR compared to AL mice, with a non-significant trend towards higher levels in CR mice, while interestingly, hepatic STAT5 phosphorylation was significantly reduced in CR compared to AL mice (**Figure 1C, D** **and Figure S3 in File S1**). The reduction in IGF-1 production in response to moderate CR is, therefore, likely via a mechanism downstream of JAK2 signaling and may be mediated, at least in part, through a disruption in STAT5 signaling.

### FGF21 expression in CR mice

Previous studies by *Inagaki et al*. and *Kubicky et al.* provide evidence that the fasting-induced hormone, FGF21, may play an important role in the down-regulation of hepatic IGF-1 production in response to CR, possibly through a STAT5-dependent mechanism [22], [28]. Given these previous findings, we sought to determine whether FGF21 expression was up-regulated in response to moderate CR (25% CR) in adult mice and, therefore, whether increased FGF21 might play a role in the IGF-1, and by extension, the cell proliferation response to this CR regimen.

**Figure 2:**
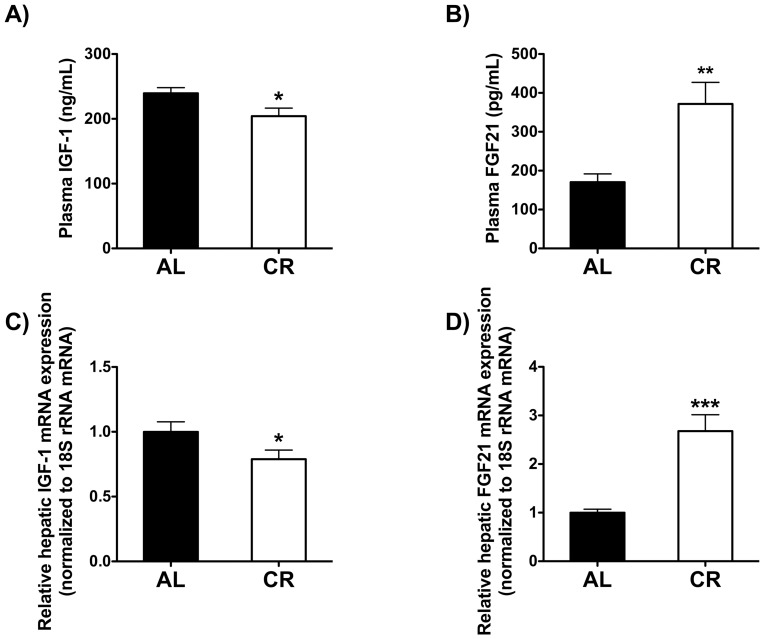
Circulating and hepatic mRNA levels of IGF-1 and FGF21 in CR mice. Plasma A) IGF-1 and B) FGF21 levels in AL and CR mice at 2100 h or 2200 h (n = 16–21 per diet). Relative hepatic mRNA expression levels of C) IGF-1 and D) FGF21 in AL and CR mice at 2100 h or 2200 h (n = 29–31 per diet). Hepatic mRNA expression levels were normalized to 18S rRNA and then normalized to the AL group. Student's unpaired two-tailed *t*-tests were used for all between-group analyses (* p<0.05, ** p<0.0043, *** p<0.0001).

Consistent with this hypothesis, we found that both circulating FGF21 and hepatic FGF21 mRNA levels were significantly increased, approximately 2.1- and 2.6-fold, respectively, in CR compared to AL mice (**Figure 2B, D**). We also confirmed in these mice that circulating IGF-1 and hepatic IGF-1 mRNA levels were significantly reduced in CR compared to AL mice, as previously observed (**Figure 2A, C**).

### FGF21 and the IGF-1 response to CR

Having confirmed that FGF21 expression is in fact up-regulated in adult mice on a moderate CR regimen, we next sought to determine whether FGF21 is necessary for the reductions in circulating IGF-1 levels and cell proliferation rates in response to moderate CR in adult mice. Toward this goal, we placed 12- to 17-week-old WT and FGF21-KO mice on either an AL or 25% CR diet for a total of 6 weeks and measured circulating IGF-1 and hepatic IGF-1 mRNA levels as well as the *in vivo* proliferation rates of keratinocytes, liver cells and splenic T-cells.

**Figure 3:**
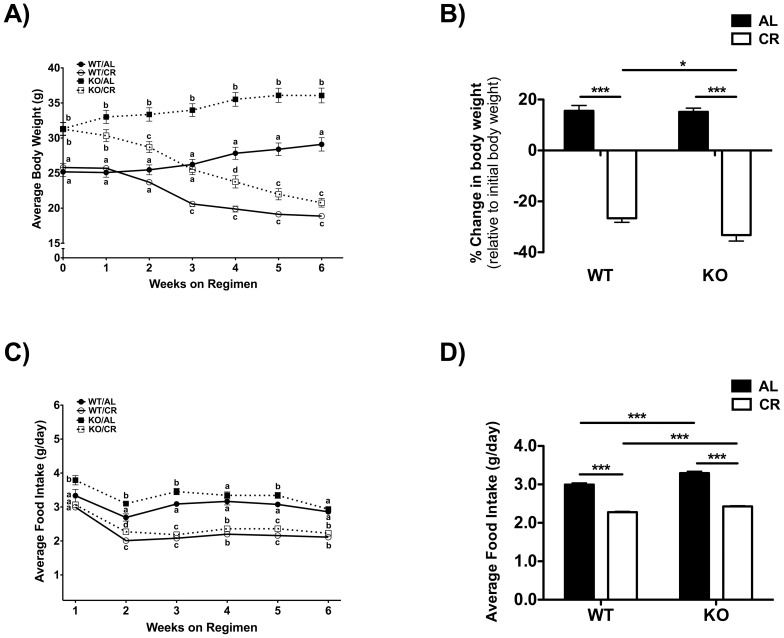
Body weight and food intake in WT and FGF21-KO mice fed AL or CR. A) Body weight trajectories and B) percent body weight change relative to initial body weight over the 6-week study. C) Daily food intake trajectories and D) cumulative average daily food intake over the 6-week study (n = 9–10 per genotype per diet). For A and C, a one-way ANOVA with a Tukey *post hoc* test was used at each time point for all between-group analyses. Groups not sharing a common letter at a given time point are significantly different (p<0.05). For B and D, a two-way ANOVA with a Bonferroni *post hoc* test was used for all between-group analyses (* p<0.05, *** p<0.001).

Consistent with previous findings, FGF21-KO mice weighed significantly more than WT mice on an AL diet [35], [37], [39], [40]. Similarly, FGF21-KO mice weighed significantly more than WT mice on a CR diet (**Figure 3A**). There was no significant difference in the percentage of weight gain over the course of the study between the two AL groups (**Figure 3B**). FGF21-KO mice, however, lost a significantly greater percentage of their body weight on a CR diet than WT mice (**Figure 3B**). On a week-by-week basis, the food intake of FGF21-KO mice tended to be higher compared to WT mice on either an AL or CR diet (**Figure 3C**). The overall average daily food intake of FGF21-KO mice was significantly higher compared to WT mice on either an AL or CR diet (**Figure 3D**).

All mice were dosed with heavy water for the last 3 weeks of the study to allow for global cell proliferation rate measurements, as previously described [38]. To take into account possible circadian fluctuations in the hormones of interest and to account for the fact that CR mice gorge their entire daily allotment of food within ∼1h of food provision, half of the mice in each group were euthanized one hour before (1500 h) and the other half three hours after (1900 h) the scheduled daily feeding of the CR mice (1600 h).

**Figure 4:**
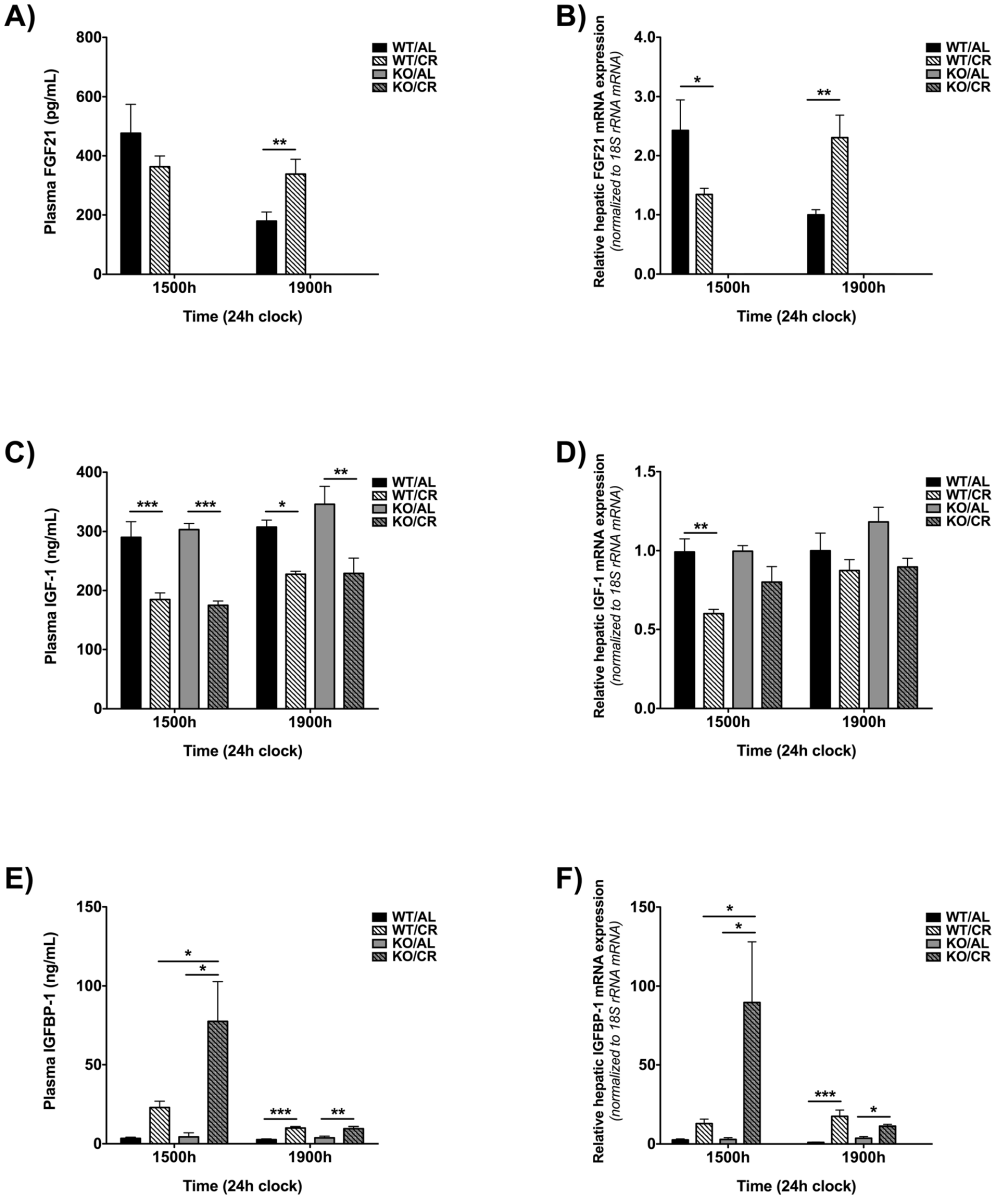
Circulating and hepatic mRNA FGF21, IGF-1 and IGFBP-1 levels in WT/AL, WT/CR, FGF21-KO/AL and FGF21-KO/CR mice. Plasma levels of A) FGF21, C) IGF-1 and E) IGFBP-1 and relative hepatic mRNA expression levels of B) FGF21, D) IGF-1 and F) IGFBP-1 at 1500 h and 1900 h in WT and FGF21-KO mice fed AL or CR (n = 3–12 per genotype per diet per time point). Hepatic mRNA expression levels were normalized to 18S rRNA and then normalized to WT/AL mice at 1500 h. All between-group analyses were performed using a two-way ANOVA with a Bonferroni *post hoc* test at each time point (* p<0.05, ** p<0.01, *** p<0.001).

Interestingly, the relative levels of circulating FGF21 and hepatic FGF21 mRNA in AL vs. CR WT mice exhibited characteristic circadian fluctuations. Compared to AL mice, circulating FGF21 and hepatic FGF21 mRNA levels trended towards being lower at 1500 h but were significantly higher at 1900 h in CR mice. FGF21 was essentially undetectable in the FGF21-KO mice (**Figure 4A, B**).

There were no significant differences in circulating IGF-1 levels between the two AL groups at either time point. Contrary to our expectations, at both time points, circulating IGF-1 levels were significantly reduced and reduced to the same extent in WT and FGF21-KO mice fed a CR diet relative to AL controls (**Figure 4C**). These changes in circulating IGF-1 levels were approximately paralleled by changes in hepatic IGF-1 mRNA expression (**Figure 4D**). These data show that FGF21 is not necessary for the reduction in IGF-1 in response to moderate CR in adult mice nor does FGF21 regulate basal IGF-1 expression in AL–fed adult mice.

Despite comparable levels of circulating IGF-1, it is possible that IGF-1 bioavailability may have differed between WT and FGF21-KO mice in response to CR. Therefore, we measured the expression of insulin-like growth factor binding protein-1 (IGFBP-1), which reduces IGF-1 bioavailability [41]–[43]. In both WT and FGF21-KO mice, CR led to a robust increase in circulating IGFBP-1 levels at both time points, although to a lesser extent at 1900 h (**Figure 4E**). While there were no significant differences in circulating IGFBP-1 levels between WT and FGF21-KO mice on a CR diet at 1900 h, these levels were significantly increased in FGF21-KO mice at 1500 h. Changes in circulating IGFBP-1 were approximately paralleled by alterations in hepatic mRNA expression (**Figure 4F**). These data show that if there is any CR-induced reduction in IGF-1 bioavailability mediated through increased IGFBP-1 expression, it is similar in both WT and FGF21-KO mice.

### FGF21 and the cell proliferation response to CR

It is important to note that IGF-1 bioavailability is influenced not only by IGFBP-1 levels, as previously mentioned, but also by a variety of other factors including acid-labile subunit (ALS), other IGFBPs and IGFBP proteases [44], [45]. Therefore, given the difficulty in assessing IGF-1 bioavailability, to determine the role of FGF21 in CR-induced changes in overall growth status, we measured *in vivo* cell proliferation rates, which serve as an integrated metric of growth stimulation.

**Figure 5:**
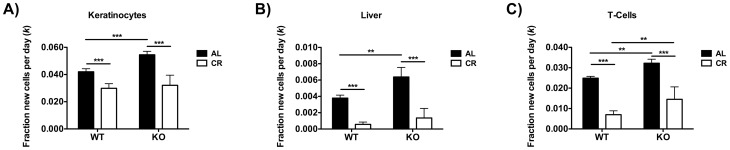
Global cell proliferation rates in WT and FGF21-KO mice fed AL or CR. Fraction of new cells per day for A) keratinocytes, B) liver cells and C) splenic T-cells during the 3 week heavy water labeling period (n = 7–10 per genotype per diet). All between-group analyses were performed using a two-way ANOVA with a Bonferroni *post hoc* test (* p<0.05, ** p<0.01, *** p<0.001).

We found that the proliferation rates of all three cell types analyzed (keratinocytes, liver cells and splenic T-cells) were significantly reduced in both WT and FGF21-KO mice fed a CR diet relative to AL controls. Interestingly, AL-fed FGF21-KO mice exhibited significantly higher rates of proliferation in all three cell types analyzed compared to AL-fed WT mice (**Figure 5A–C**), despite similar circulating IGF-1 and IGFBP-1 levels. It is possible that these higher rates of cell proliferation may be related to higher food intake in AL-fed FGF21-KO compared to AL-fed WT mice. Overall these data show that FGF21 is not necessary for the reduction in global cell proliferation rates in response to moderate CR and indirectly suggest that FGF21 may reduce basal cell proliferation rates under normal, AL feeding conditions.

## Discussion

In the context of moderate CR in adult mice, the studies presented here demonstrate the following: 1) Circulating GH and hepatic JAK2 phosphorylation levels are unchanged in CR compared to AL-fed WT mice, therefore, changes in factors within the hepatic GH-mediated IGF-1 production pathway upstream of JAK2 cannot account for the reduction in IGF-1 observed in response to moderate CR. 2) Hepatic STAT5 phosphorylation levels are reduced in CR compared to AL-fed WT mice and may in part mediate the reduction in IGF-1 in these mice. 3) The level of FGF21 expression in AL vs. CR WT mice displays a characteristic circadian pattern with FGF21 expression being lower at 1500 h and higher at 1900 h in CR compared to AL mice. 4) Ablation of FGF21 has no effect on CR-induced reductions in IGF-1 levels or cell proliferation rates.

Under AL feeding conditions, circulating IGF-1 levels are primarily determined by GH signaling in the liver [19], [20]. We report here, to our knowledge for the first time, GH secretory dynamics and hepatic GH signaling in mice on a moderate CR regimen. Contrary to findings in CR rats, in which circulating GH levels are reduced, and in mice on severe CR (50–60% CR), in which circulating GH levels are increased, we found that circulating GH levels were unchanged in mice on moderate CR compared to AL controls [11], [22], [23], [25], [46], [47]. It has also been previously reported that severe CR (50% CR) in mice reduces hepatic GH binding and GHR expression [22]. While we did not directly measure hepatic GH binding or GHR expression in the present studies, we did find that hepatic JAK2 phosphorylation was unchanged while hepatic STAT5 phosphorylation was reduced in mice on moderate CR compared to AL controls. These findings are significant in that they suggest that the reduction in IGF-1 in response to this CR regimen is likely via a mechanism downstream of JAK2 signaling and may be mediated through a disruption in STAT5 signaling. Our results rule out the possibility that several mechanism(s) upstream of and including reduced hepatic JAK2 activity may mediate the observed reductions in GH-mediated hepatic STAT5 phosphorylation and IGF-1 levels in mice on moderate CR including, 1) reduced GHR expression, 2) increased Leptin Receptor Overlapping Transcript (LEPROT) and/or LEPROT-like 1 (LEPROTL1) expression, which have been shown to reduce cell-surface GHR content in hepatocytes as well as shown to mediate the FGF21-induced reduction in hepatic GH binding in mice on severe CR (50% CR) [29], [48], 3) increased expression of the protein tyrosine phosphatases (PTP) PTP-1b and SHP-1 which have been shown to dephosphorylate the GH-activated form of JAK2 as well as to dephosphorylate GHR itself in the case of PTP-1b [49] and 4) increased expression of suppressor of cytokine signaling 2 (SOCS2), which has been shown to promote proteasomal degradation of GHR [49]. However, SOCS2 has also been shown to bind phospho-tyrosine residues on GHR, thereby, competitively inhibiting STAT5 binding to GHR, thus inhibiting STAT5 phosphorylation and activation [49] so it is still possible that SOCS2 may play a role in the reduction of STAT5 phosphorylation in mice on moderate CR.

Previous findings in mice by *Inagaki et al.* implicated FGF21 as a negative regulator of IGF-1 expression via a mechanism downstream of JAK2 signaling (possibly involving SOCS2) while *Kuhla et al.* and *Kubicky et al.* report that FGF21 expression is increased in mice on a long-term CR diet (74wks) and in young mice on a severe CR diet (50% CR) compared to AL controls, respectively [22], [28], [31]. Consistent with the findings of *Kuhla et al.* and *Kubicky et al.*, we found that circulating FGF21 and hepatic FGF21 mRNA levels were increased in adult mice on a moderate CR diet compared to AL-fed controls when measured ∼5h after food provision to CR mice (**Figure 2**). In light of the findings of *Inagaki et al., Kuhla et al.* and *Kubicky et al*., our findings that FGF21 expression is up-regulated in CR mice (at the initial time point assessed) and that hepatic GH signaling is blunted downstream of JAK2 in CR mice, led us to hypothesize that FGF21 may mediate the reduction in IGF-1 and, therefore, the reduction in cell proliferation rates, in response to moderate CR.

Using FGF21-KO mice, we asked directly whether FGF21 was necessary for the IGF-1 and cell proliferation responses to moderate CR in adult mice. In order to capture possible circadian fluctuations in mRNA and circulating levels of factors of interest, mice were euthanized at two different time points. We found that the relative levels of circulating FGF21 and hepatic FGF21 mRNA in AL vs. CR WT mice exhibited characteristic circadian fluctuations (**Figure 4A, B**). The pattern of FGF21 expression in response to CR was unexpected in light of previous observations that FGF21 expression is robustly up-regulated in fasted mice [33]–[35]. In contrast to these studies, we found that at 1500 h, CR mice, which had essentially been without food for more than 20 h, had lower hepatic FGF21 mRNA levels and a trend towards lower circulating levels of FGF21 compared to AL mice. Furthermore, at 1900 h, CR mice, which were in a postprandial state, had higher circulating FGF21 and hepatic FGF21 mRNA levels compared to AL mice. These data underscore the fact that CR is not simply repeated fasting and that CR and fasting are two distinct dietary paradigms. Our observation that hepatic FGF21 mRNA levels are up-regulated in CR mice upon feeding is not without precedent as a previous report demonstrated that refeeding after a fast robustly increases circulating FGF21 and hepatic FGF21 mRNA levels in rats [50]. Together these data underscore the critical importance of the timing of measurements in the context of AL, as well as non-AL feeding.

This critical timing effect was highlighted in a previous study from our group with regard to hepatic expression of genes involved in *de novo lipogenesis* (DNL) [36]. With regard to FGF21 expression, it is possible that the apparent absence of any effect of CR on FGF21 expression in a subset of previous studies in rodents is due to the timing of tissue collection relative to the time of food provision to CR animals. Consistent with the findings presented here, these previous studies found that FGF21 levels tended to be comparable or reduced in CR compared to AL rodents when tissues were collected ∼20 h after food provision to CR animals [30], [51].

Despite being higher at 1900 h in CR compared to AL WT mice, we found by studying FGF21-KO mice that FGF21 was not required for the reduction in circulating IGF-1 in response to moderate CR (**Figure 4D**). This finding was unexpected given reports that exogenous administration of recombinant FGF21 and overexpression of FGF21 in mice (resulting in a >14-fold increase in circulating FGF21 levels), reduces IGF-1 expression [22], [28]. Our finding that FGF21 is not required for the reduction in IGF-1 in response to CR is also contrary to a report that FGF21 is necessary for the full reduction in circulating IGF-1 in response to undernutrition in mice [22]. In this previous study, young (4-week-old) developing mice were placed on a severe CR diet (50% CR) for 4 weeks while in the present study, adult (12- to 17-week-old) mice were placed on a moderate CR diet (25% CR) for 6 weeks. There are several potential explanations for the differences between the results of these above-mentioned previous studies and those of the present study, including: i) There may be a threshold of FGF21 concentration above which its regulatory effects on IGF-1 expression manifest, ii) There may be a threshold of %CR above which the regulatory effects of FGF21 on IGF-1 expression manifest and iii) FGF21 may regulate IGF-1 expression primarily during development in WT mice. Clearly the relationship between FGF21 and IGF-1 expression and nutritional status in mice is complex. Future studies should focus on other factors that may regulate changes in IGF-1 expression in response to moderate short-term CR in adult WT mice, including protein intake, insulin and thyroid hormone [24].

Interestingly in humans, long-term (1–6y) CR does not reduce circulating IGF-1 levels and a very low calorie diet in obese diabetics actually reduces circulating FGF21 levels, with the caveat that baseline circulating FGF21 levels are elevated in this population [52], [53]. More studies are needed to confirm and clarify the effect of varying degrees of CR on circulating IGF-1 and FGF21 levels and the potential interplay between these two hormones in healthy humans.

In the context of moderate CR in mice, the present study provides evidence that FGF21 is not necessary for the CR-induced reduction in circulating IGF-1 levels. In addition, our finding that circulating IGF-1 levels were significantly reduced in FGF21-KO/CR compared to FGF21-KO/AL mice at 1900 h while hepatic STAT5 phosphorylation was if anything elevated in FGF21-KO/CR compared to FGF21-KO/AL at this same time point (**Figures S4 and S5 in File S1**), provides evidence that STAT5 is not the sole regulator of IGF-1 production.

We also show in the present study that FGF21 is not required for the reduction in cell proliferation rates in response to moderate CR (**Figure 5**). An unexpected finding was that despite similar circulating IGF-1 and IGFBP-1 levels, AL-fed FGF21-KO mice exhibit higher rates of proliferation in all three cell types analyzed compared to AL-fed WT mice. These data are consistent with the reduced susceptibility of transgenic mice over-expressing FGF21 in hepatocytes to diethylnitrosamine (DEN)-induced liver tumor formation [54]. This higher rate of cell proliferation in AL-fed FGF21-KO compared to WT mice suggests that FGF21 may have direct or indirect anti-anabolic/anti-mitotic effects that are independent of circulating IGF-1. Alternatively, the higher rate of cell proliferation in AL-fed FGF21-KO compared to WT mice may be related to higher food intake.

Despite the lack of an effect of FGF21 on the IGF-1 and cell proliferation responses to moderate CR in adult mice, FGF21 may have an important role in other responses to this CR regimen. FGF21 is highly expressed in the pancreas and has well-established glucose-lowering and insulin-sensitizing effects *in vivo* [26], [27], [37], [55]–[59]. It will be interesting to determine what if any role FGF21 plays in the glucose-lowering and insulin-sensitizing effects of CR [60]–[62].

In conclusion, we have shown here that moderate CR in adult mice results in GH resistance downstream of JAK2 signaling and that the relative expression of FGF21 in AL vs. CR mice exhibits a characteristic circadian pattern. We have also shown that FGF21 is not necessary for the reductions in IGF-1 or cell proliferation rates in response to moderate CR. Finally, in the context of AL feeding, our data indirectly suggest that FGF21 may have anti-anabolic/anti-mitotic effects.

While FGF21 does not appear to be necessary for the reduction in circulating IGF-1 in response to moderate CR in adult mice, there is evidence that FGF21 is sufficient to reduce circulating IGF-1 levels in mice when exogenously administered and when overexpressed [22], [28]. Given these observations in conjunction with its glucose-lowering and insulin-sensitizing effects, FGF21 appears to mimic several of the beneficial effects of CR, underscoring its therapeutic potential.

## Supporting Information

File S1 Contains the following files: **Figure S1.** Time course of plasma GH levels in CR and AL mice. Plasma GH levels in A) AL and B) CR mice over 5.25 h time course experiment (n = 8 per diet). Blood was collected from each mouse via the tail vein every 45 min. Each line represents the plasma GH levels over the time course experiment for a single mouse. CR mice were fed at their regular feeding time (1600 h). **Figure S2.** Time course of plasma GH levels in CR and AL mice. Plasma GH levels in AL and CR mice at A) 1530 h, B) 1615 h, C) 1700 h, D) 174 5h, E) 1830 h, F) 1915 h, G) 2000 h and H) 2045 h (n = 8 per diet). Blood was collected from each mouse via the tail vein every 45 min. CR mice were fed at their regular feeding time (1600h). Student's unpaired two-tailed t-tests were used for all between-group analyses. **Figure S3.** Blots used to quantify hepatic ph-total JAK2 and STAT5 levels in AL and CR mice. Hepatic protein levels of A–B) Total-JAK2, C–D) ph-JAK2, E–F) Total-STAT5 and G–H) ph-STAT5. * = One or more bands associated with ph/total quantification was not assessed or was imperfect. Blots were cut at indicated locations prior to primary antibody incubation. **Figure S4.** Ph-total hepatic STAT5 levels in AL-fed and CR-fed WT and FGF21-KO mice. n = 4–5 per genotype per diet per time point. Data normalized to WT/AL mice at 1500 h. All between-group analyses were performed using a two-way ANOVA with a Bonferroni post hoc test at each time point. **Figure S5.** Blots used to quantify hepatic ph-total STAT5 levels in AL-fed and CR-fed WT and FGF21-KO mice. Hepatic protein levels of A) Total-STAT5 in WT/AL and WT/CR mice at 1500 h, B) ph-STAT5 in WT/AL and WT/CR mice at 1500 h, C) Total-STAT5 in KO/AL and KO/CR mice at 1500 h, D) ph-STAT5 in KO/AL and KO/CR mice at 1500 h, E) Total-STAT5 in WT/AL and WT/CR mice at 1900 h, F) ph-STAT5 in WT/AL and WT/CR mice at 1900 h, G) Total-STAT5 in KO/AL and KO/CR mice at 1900 h and H) ph-STAT5 in KO/AL and KO/CR mice at 1900h. + con  =  extract from UT-7 cells treated with GM-CSF (positive control for STAT5 phosphorylation). Blots were cut at indicated locations prior to primary antibody incubation.
(PDF)

## References

[1] Katewa SD, Kapahi P (2010) Dietary restriction and aging, 2009. Aging Cell 9: 105–112 10.1111/j.1474-9726.2010.00552.x 20096035 PMC2958258

[2] Kemnitz JW (2011) Calorie restriction and aging in nonhuman primates. ILAR J 52: 66–77. 21411859 10.1093/ilar.52.1.66 PMC3278796

[3] Sonntag WE, Lynch CD, Cefalu WT, Ingram RL, Bennett SA, et al (1999) Pleiotropic effects of growth hormone and insulin-like growth factor (IGF)-1 on biological aging: inferences from moderate caloric-restricted animals. J Gerontol A Biol Sci Med Sci 54: B521–B538. 10647962 10.1093/gerona/54.12.b521

[4] De Magalhães JP, Faragher RGA (2008) Cell divisions and mammalian aging: integrative biology insights from genes that regulate longevity. Bioessays 30: 567–578 10.1002/bies.20760 18478536

[5] Lok E, Nera EA, Iverson F, Scott F, So Y, et al (1988) Dietary restriction, cell proliferation and carcinogenesis: a preliminary study. Cancer Lett 38: 249–255. 3349445 10.1016/0304-3835(88)90016-x

[6] Lok E, Scott FW, Mongeau R, Nera EA, Malcolm S, et al (1990) Calorie restriction and cellular proliferation in various tissues of the female Swiss Webster mouse. Cancer Lett 51: 67–73. 2337899 10.1016/0304-3835(90)90232-m

[7] Wolf NS, Penn PE, Jiang D, Fei RG, Pendergrass WR (1995) Caloric restriction: conservation of in vivo cellular replicative capacity accompanies life-span extension in mice. Exp Cell Res 217: 317–323 10.1006/excr.1995.1092 7698231

[8] Bruss MD, Thompson ACS, Aggarwal I, Khambatta CF, Hellerstein MK (2011) The effects of physiological adaptations to calorie restriction on global cell proliferation rates. Am J Physiol Endocrinol Metab 300: E735–E745 10.1152/ajpendo.00661.2010 21285400 PMC3279299

[9] Hsieh EA, Chai CM, Hellerstein MK (2005) Effects of caloric restriction on cell proliferation in several tissues in mice: role of intermittent feeding. Am J Physiol Endocrinol Metab 288: E965–E972 10.1152/ajpendo.00368.2004 15613681

[10] Mendenhall CL, Roselle GA, Gartside P, Grossman CJ (1997) Effects of recombinant human insulin-like growth factor-1 and recombinant human growth hormone on anabolism and immunity in calorie-restricted alcoholic rats. Alcohol Clin Exp Res 21: 1–10. 9046366

[11] Hursting SD, Switzer BR, French JE, Kari FW (1993) The growth hormone: insulin-like growth factor 1 axis is a mediator of diet restriction-induced inhibition of mononuclear cell leukemia in Fischer rats. Cancer Res 53: 2750–2757. 8389243

[12] Dunn SE, Kari FW, French J, Leininger JR, Travlos G, et al (1997) Dietary restriction reduces insulin-like growth factor I levels, which modulates apoptosis, cell proliferation, and tumor progression in p53-deficient mice. Cancer Res 57: 4667–4672. 9354418

[13] Bielschowsky F, Bielschowsky M (1961) Carcinogenesis in the Pituitary Dwarf Mouse. The Response to Dimethylbenzanthracene Applied to the Skin. Br J Cancer 15: 257–262. 21772452 10.1038/bjc.1961.32 PMC2070912

[14] Ikeno Y, Bronson RT, Hubbard GB, Lee S, Bartke A (2003) Delayed occurrence of fatal neoplastic diseases in ames dwarf mice: correlation to extended longevity. J Gerontol A Biol Sci Med Sci 58: 291–296. 12663691 10.1093/gerona/58.4.b291

[15] Alderman JM, Flurkey K, Brooks NL, Naik SB, Gutierrez JM, et al (2009) Neuroendocrine inhibition of glucose production and resistance to cancer in dwarf mice. Exp Gerontol 44: 26–33 10.1016/j.exger.2008.05.014 18582556 PMC2872123

[16] Ikeno Y, Hubbard GB, Lee S, Cortez LA, Lew CM, et al (2009) Reduced incidence and delayed occurrence of fatal neoplastic diseases in growth hormone receptor/binding protein knockout mice. J Gerontol A Biol Sci Med Sci 64: 522–529 10.1093/gerona/glp017 19228785 PMC2667132

[17] Flurkey K, Papaconstantinou J, Miller RA, Harrison DE (2001) Lifespan extension and delayed immune and collagen aging in mutant mice with defects in growth hormone production. Proc Natl Acad Sci USA 98: 6736–6741 10.1073/pnas.111158898 11371619 PMC34422

[18] Liang H, Masoro EJ, Nelson JF, Strong R, McMahan CA, et al (2003) Genetic mouse models of extended lifespan. Exp Gerontol 38: 1353–1364. 14698816 10.1016/j.exger.2003.10.019

[19] Yakar S, Liu JL, Stannard B, Butler A, Accili D, et al (1999) Normal growth and development in the absence of hepatic insulin-like growth factor I. Proc Natl Acad Sci USA. 96: 7324–7329. 10.1073/pnas.96.13.7324 PMC22084 10377413

[20] Rosenfeld RG, Hwa V (2009) The growth hormone cascade and its role in mammalian growth. Horm Res 71 Suppl 236–40 10.1159/000192434 19407495

[21] Herrington J, Smit LS, Schwartz J, Carter-Su C (2000) The role of STAT proteins in growth hormone signaling. Oncogene 19: 2585–2597 10.1038/sj.onc.1203526 10851057

[22] Kubicky RA, Wu S, Kharitonenkov A, De Luca F (2012) Role of fibroblast growth factor 21 (FGF21) in undernutrition-related attenuation of growth in mice. Endocrinology 153: 2287–2295 10.1210/en.2011-1909 22374976

[23] Zhao T-J, Liang G, Li RL, Xie X, Sleeman MW, et al (2010) Ghrelin O-acyltransferase (GOAT) is essential for growth hormone-mediated survival of calorie-restricted mice. Proc Natl Acad Sci USA 107: 7467–7472 10.1073/pnas.1002271107 20231469 PMC2867684

[24] Thissen JP, Ketelslegers JM, Underwood LE (1994) Nutritional regulation of the insulin-like growth factors. Endocr Rev 15: 80–101 10.1210/edrv-15-1-80 8156941

[25] Sonntag WE, Xu X, Ingram RL, D'Costa A (1995) Moderate caloric restriction alters the subcellular distribution of somatostatin mRNA and increases growth hormone pulse amplitude in aged animals. Neuroendocrinology 61: 601–608. 7617139 10.1159/000126885

[26] Kharitonenkov A, Shiyanova TL, Koester A, Ford AM, Micanovic R, et al (2005) FGF-21 as a novel metabolic regulator. J Clin Invest 115: 1627–1635 10.1172/JCI23606 15902306 PMC1088017

[27] Fon Tacer K, Bookout AL, Ding X, Kurosu H, John GB, et al (2010) Research resource: Comprehensive expression atlas of the fibroblast growth factor system in adult mouse. Mol Endocrinol 24: 2050–2064 10.1210/me.2010-0142 20667984 PMC2954642

[28] Inagaki T, Lin VY, Goetz R, Mohammadi M, Mangelsdorf DJ, et al (2008) Inhibition of growth hormone signaling by the fasting-induced hormone FGF21. Cell Metab 8: 77–83 10.1016/j.cmet.2008.05.006 18585098 PMC2575072

[29] Wu S, Grunwald T, Kharitonenkov A, Dam J, Jockers R, et al (2013) Increased expression of fibroblast growth factor 21 (FGF21) during chronic undernutrition causes growth hormone insensitivity in chondrocytes by inducing leptin receptor overlapping transcript (LEPROT) and leptin receptor overlapping transcript-like 1 (LEPROTL1) expression. J Biol Chem 288: 27375–27383 10.1074/jbc.M113.462218 23940039 PMC3779732

[30] Zhang Y, Xie Y, Berglund ED, Coate KC, He TT, et al (2012) The starvation hormone, fibroblast growth factor-21, extends lifespan in mice. Elife 1: e00065 10.7554/eLife.00065 23066506 PMC3466591

[31] Kuhla A, Hahn S, Butschkau A, Lange S, Wree A, et al.. (2013) Lifelong Caloric Restriction Reprograms Hepatic Fat Metabolism in Mice. J Gerontol A Biol Sci Med Sci. doi:10.1093/gerona/glt160. 10.1093/gerona/glt160 24149425

[32] Lundåsen T, Hunt MC, Nilsson L-M, Sanyal S, Angelin B, et al (2007) PPARalpha is a key regulator of hepatic FGF21. Biochem Biophys Res Commun 360: 437–440 10.1016/j.bbrc.2007.06.068 17601491

[33] Inagaki T, Dutchak P, Zhao G, Ding X, Gautron L, et al (2007) Endocrine regulation of the fasting response by PPARalpha-mediated induction of fibroblast growth factor 21. Cell Metab 5: 415–425 10.1016/j.cmet.2007.05.003 17550777

[34] Badman MK, Pissios P, Kennedy AR, Koukos G, Flier JS, et al (2007) Hepatic fibroblast growth factor 21 is regulated by PPARalpha and is a key mediator of hepatic lipid metabolism in ketotic states. Cell Metab 5: 426–437 10.1016/j.cmet.2007.05.002 17550778

[35] Hotta Y, Nakamura H, Konishi M, Murata Y, Takagi H, et al (2009) Fibroblast growth factor 21 regulates lipolysis in white adipose tissue but is not required for ketogenesis and triglyceride clearance in liver. Endocrinology 150: 4625–4633 10.1210/en.2009-0119 19589869

[36] Bruss MD, Khambatta CF, Ruby MA, Aggarwal I, Hellerstein MK (2010) Calorie restriction increases fatty acid synthesis and whole body fat oxidation rates. Am J Physiol Endocrinol Metab 298: E108–E116 10.1152/ajpendo.00524.2009 19887594 PMC4056782

[37] Badman MK, Koester A, Flier JS, Kharitonenkov A, Maratos-Flier E (2009) Fibroblast growth factor 21-deficient mice demonstrate impaired adaptation to ketosis. Endocrinology 150: 4931–4940 10.1210/en.2009-0532 19819944 PMC2775979

[38] Busch R, Neese RA, Awada M, Hayes GM, Hellerstein MK (2007) Measurement of cell proliferation by heavy water labeling. Nat Protoc 2: 3045–3057 10.1038/nprot.2007.420 18079703

[39] Fisher FM, Chui PC, Antonellis PJ, Bina HA, Kharitonenkov A, et al (2010) Obesity is a fibroblast growth factor 21 (FGF21)-resistant state. Diabetes 59: 2781–2789 10.2337/db10-0193 20682689 PMC2963536

[40] Adams AC, Coskun T, Cheng CC, O Farrell LS, Dubois SL, et al (2013) Fibroblast growth factor 21 is not required for the antidiabetic actions of the thiazoladinediones. Mol Metab 2: 205–214 10.1016/j.molmet.2013.05.005 24049735 PMC3773835

[41] Ferry RJ Jr, Cerri RW, Cohen P (1999) Insulin-like growth factor binding proteins: new proteins, new functions. Horm Res 51: 53–67 doi:23315 10352394 10.1159/000023315

[42] McCarty MF (1997) Up-regulation of IGF binding protein-1 as an anticarcinogenic strategy: relevance to caloric restriction, exercise, and insulin sensitivity. Med Hypotheses 48: 297–308. 9160283 10.1016/s0306-9877(97)90098-0

[43] Sandhu MS, Dunger DB, Giovannucci EL (2002) Insulin, insulin-like growth factor-I (IGF-I), IGF binding proteins, their biologic interactions, and colorectal cancer. J Natl Cancer Inst 94: 972–980. 12096082 10.1093/jnci/94.13.972

[44] Boisclair YR, Rhoads RP, Ueki I, Wang J, Ooi GT (2001) The acid-labile subunit (ALS) of the 150 kDa IGF-binding protein complex: an important but forgotten component of the circulating IGF system. J Endocrinol 170: 63–70. 11431138 10.1677/joe.0.1700063

[45] Lelbach A, Muzes G, Feher J (2005) The insulin-like growth factor system: IGFs, IGF-binding proteins and IGFBP-proteases. Acta Physiol Hung 92: 97–107 10.1556/APhysiol.92.2005.2.1 16268048

[46] Chacón F, Esquifino AI, Perelló M, Cardinali DP, Spinedi E, et al (2005) 24-hour changes in ACTH, corticosterone, growth hormone, and leptin levels in young male rats subjected to calorie restriction. Chronobiol Int 22: 253–265. 16021842 10.1081/cbi-200053522

[47] Armario A, Montero JL, Jolin T (1987) Chronic food restriction and the circadian rhythms of pituitary-adrenal hormones, growth hormone and thyroid-stimulating hormone. Ann Nutr Metab 31: 81–87. 3035995 10.1159/000177254

[48] Touvier T, Conte-Auriol F, Briand O, Cudejko C, Paumelle R, et al (2009) LEPROT and LEPROTL1 cooperatively decrease hepatic growth hormone action in mice. J Clin Invest 119: 3830–3838 10.1172/JCI34997 19907080 PMC2786784

[49] Herington AC, Lobie PE (2012) Signal Transduction Mechanisms Underlying Growth Hormone Receptor Action. The Open Endocrinology Journal 6: 13–21.

[50] Sánchez J, Palou A, Picó C (2009) Response to carbohydrate and fat refeeding in the expression of genes involved in nutrient partitioning and metabolism: striking effects on fibroblast growth factor-21 induction. Endocrinology 150: 5341–5350 10.1210/en.2009-0466 19837871

[51] Sharma N, Castorena CM, Cartee GD (2012) Greater insulin sensitivity in calorie restricted rats occurs with unaltered circulating levels of several important myokines and cytokines. Nutr Metab (Lond) 9: 90 10.1186/1743-7075-9-90 23067400 PMC3541154

[52] Fontana L, Weiss EP, Villareal DT, Klein S, Holloszy JO (2008) Long-term effects of calorie or protein restriction on serum IGF-1 and IGFBP-3 concentration in humans. Aging Cell 7: 681–687. 18843793 10.1111/j.1474-9726.2008.00417.x PMC2673798

[53] Lips MA, de Groot GH, Berends FJ, Wiezer R, van Wagensveld BA, et al.. (2014) Calorie restriction and Roux-en-Y gastric bypass have opposing effects on circulating FGF21 in morbidly obese subjects. Clin Endocrinol (Oxf). doi:10.1111/cen.12496. 10.1111/cen.12496 24841294

[54] Huang X, Yu C, Jin C, Yang C, Xie R, et al (2006) Forced expression of hepatocyte-specific fibroblast growth factor 21 delays initiation of chemically induced hepatocarcinogenesis. Mol Carcinog 45: 934–942 10.1002/mc.20241 16929488

[55] Coskun T, Bina HA, Schneider MA, Dunbar JD, Hu CC, et al (2008) Fibroblast growth factor 21 corrects obesity in mice. Endocrinology 149: 6018–6027 10.1210/en.2008-0816 18687777

[56] Xu J, Stanislaus S, Chinookoswong N, Lau YY, Hager T, et al. (2009) Acute glucose-lowering and insulin-sensitizing action of FGF21 in insulin resistant mouse models – Association with liver and adipose tissue effects. Am J Physiol Endocrinol Metab. Available: http://www.ncbi.nlm.nih.gov/pubmed/19706786. Accessed 24 May 2011. 10.1152/ajpendo.00348.2009 19706786

[57] Sarruf DA, Thaler JP, Morton GJ, German J, Fischer JD, et al (2010) Fibroblast growth factor 21 action in the brain increases energy expenditure and insulin sensitivity in obese rats. Diabetes 59: 1817–1824 10.2337/db09-1878 20357365 PMC2889784

[58] Xu J, Lloyd DJ, Hale C, Stanislaus S, Chen M, et al (2009) Fibroblast growth factor 21 reverses hepatic steatosis, increases energy expenditure, and improves insulin sensitivity in diet-induced obese mice. Diabetes 58: 250–259 10.2337/db08-0392 18840786 PMC2606881

[59] Berglund ED, Li CY, Bina HA, Lynes SE, Michael MD, et al (2009) Fibroblast growth factor 21 controls glycemia via regulation of hepatic glucose flux and insulin sensitivity. Endocrinology 150: 4084–4093 10.1210/en.2009-0221 19470704 PMC2736088

[60] McKee Alderman J, DePetrillo MA, Gluesenkamp AM, Hartley AC, Verhoff SV, et al (2010) Calorie restriction and dwarf mice in gerontological research. Gerontology 56: 404–409 10.1159/000235720 19690401

[61] Masoro EJ (2005) Overview of caloric restriction and ageing. Mech Ageing Dev 126: 913–922 10.1016/j.mad.2005.03.012 15885745

[62] Heilbronn LK, Ravussin E (2003) Calorie restriction and aging: review of the literature and implications for studies in humans. Am J Clin Nutr 78: 361–369. 12936916 10.1093/ajcn/78.3.361

