# Supplementary material for: Fibroblast Growth Factor 21 Is Not Required for the Reductions in Circulating Insulin-Like Growth Factor-1 or Global Cell Proliferation Rates in Response to Moderate Calorie Restriction in Adult Mice
Source: PLoS One. 2014 Nov 4;9(11):e111418. doi: 10.1371/journal.pone.0111418 (PMC4219748; doi:10.1371/journal.pone.0111418)
Supplement: File S1 — Contains the following files: Figure S1. Time course of plasma GH levels in CR and AL mice. Plasma GH levels in A) AL and B) CR mice over 5.25 h time course experiment (n = 8 per diet). Blood was collected from each mouse via the tail vein every 45 min. Each line represents the plasma GH levels over the time course experiment for a single mouse. CR mice were fed at their regular feeding time (1600 h). Figure S2. Time course of plasma GH levels in CR and AL mice. Plasma GH levels in AL and CR mice at A) 1530 h, B) 1615 h, C) 1700 h, D) 174 5h, E) 1830 h, F) 1915 h, G) 2000 h and H) 2045 h (n = 8 per diet). Blood was collected from each mouse via the tail vein every 45 min. CR mice were fed at their regular feeding time (1600h). Student's unpaired two-tailed t-tests were used for all between-group analyses. Figure S3. Blots used to quantify hepatic ph-total JAK2 and STAT5 levels in AL and CR mice. Hepatic protein levels of A–B) Total-JAK2, C–D) ph-JAK2, E–F) Total-STAT5 and G–H) ph-STAT5. * = One or more bands associated with ph/total quantification was not assessed or was imperfect. Blots were cut at indicated locations prior to primary antibody incubation. Figure S4. Ph-total hepatic STAT5 levels in AL-fed and CR-fed WT and FGF21-KO mice. n = 4–5 per genotype per diet per time point. Data normalized to WT/AL mice at 1500 h. All between-group analyses were performed using a two-way ANOVA with a Bonferroni post hoc test at each time point. Figure S5. Blots used to quantify hepatic ph-total STAT5 levels in AL-fed and CR-fed WT and FGF21-KO mice. Hepatic protein levels of A) Total-STAT5 in WT/AL and WT/CR mice at 1500 h, B) ph-STAT5 in WT/AL and WT/CR mice at 1500 h, C) Total-STAT5 in KO/AL and KO/CR mice at 1500 h, D) ph-STAT5 in KO/AL and KO/CR mice at 1500 h, E) Total-STAT5 in WT/AL and WT/CR mice at 1900 h, F) ph-STAT5 in WT/AL and WT/CR mice at 1900 h, G) Total-STAT5 in KO/AL and KO/CR mice at 1900 h and H) ph-STAT5 in KO/AL and KO/CR mice at 1900h. + con = extract [file pone.0111418.s001.pdf]

Supporting Information

Fibroblast growth factor 21 is not required for the reductions in circulating insulin-like growth factor-1 or global cell proliferation rates in response to moderate calorie restriction in adult mice

By Airlia C. S. Thompson, Matthew D. Bruss, Nitish Nag, Alexei Kharitonenkov, Andrew C. Adams, Marc K. Hellerstein

Supporting information figures S1-S5:

Figure S1

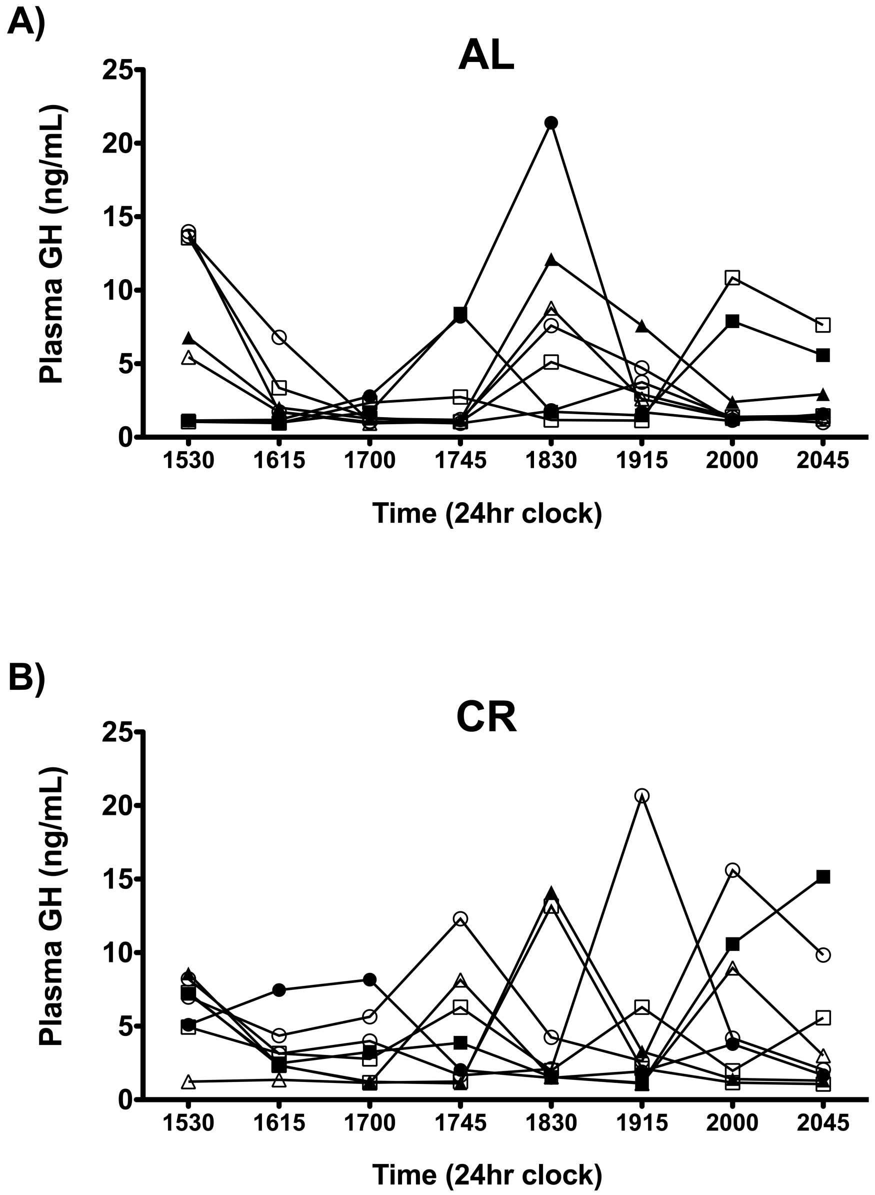

Figure S1. Time course of plasma GH levels in CR and AL mice. Plasma GH levels in A) AL and B) CR mice over 5.25h time course experiment (n = 8 per diet). Blood was collected from each mouse via the tail vein every 45min. Each line represents the plasma GH levels over the time course experiment for a single mouse. CR mice were fed at their regular feeding time (1600h).

Figure S2

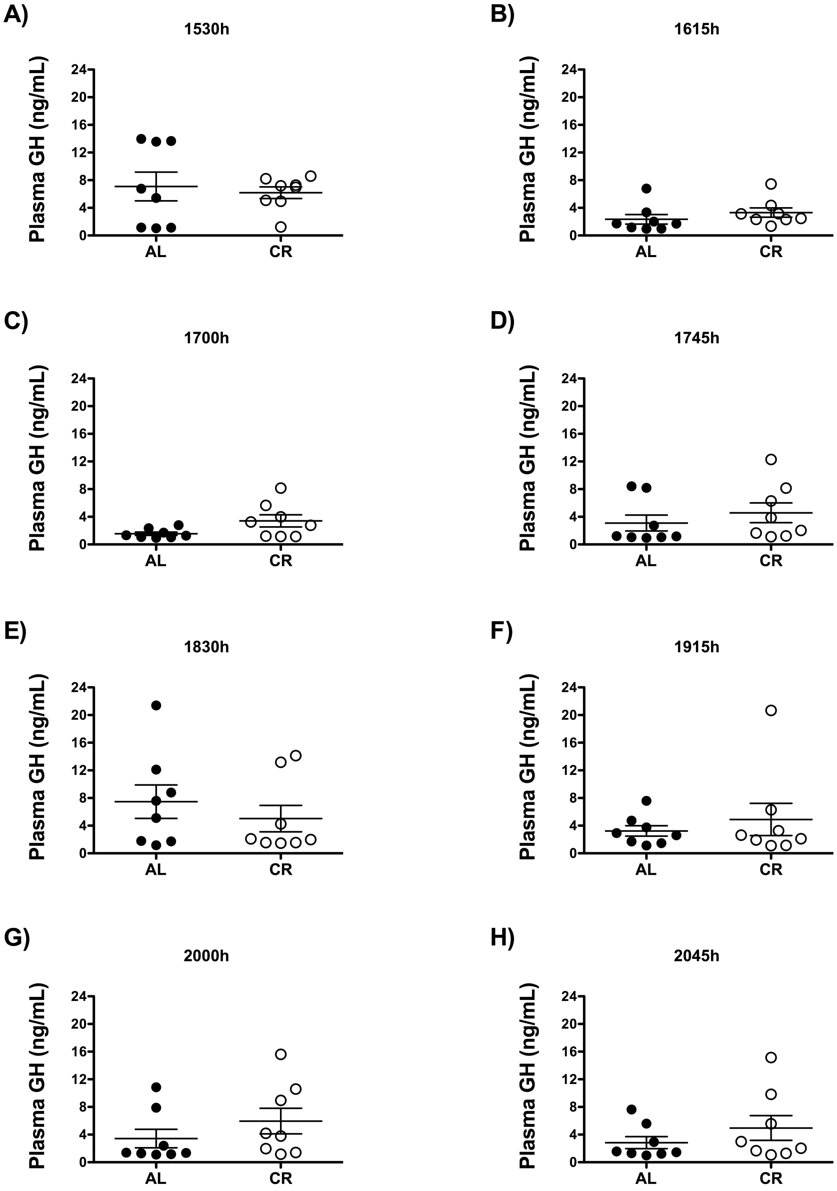

Figure S2. Time course of plasma GH levels in CR and AL mice. Plasma GH levels in AL and CR mice at A) 1530h, B) 1615h, C) 1700h, D) 1745h, E) 1830h, F) 1915h, G) 2000h and H) 2045h (n = 8 per diet). Blood was collected from each mouse via the tail vein every 45min. CR mice were fed at their regular feeding time (1600h). Student's unpaired two-tailed *t*-tests were used for all between-group analyses.

Figure S3

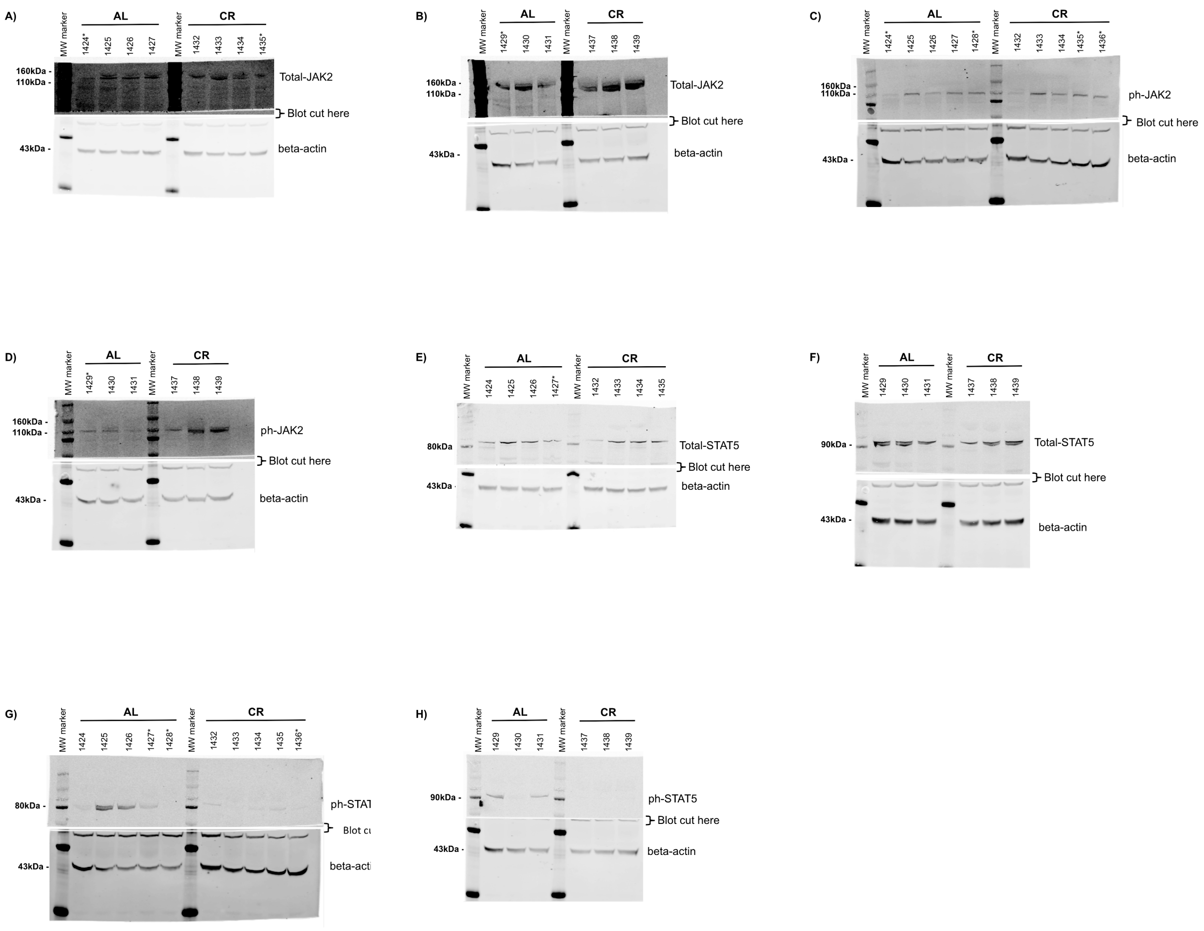

Figure S3. Blots used to quantify hepatic ph-total JAK2 and STAT5 levels in AL and CR mice. Hepatic protein levels of A-B) Total-JAK2, C-D) ph-JAK2, E-F) Total-STAT5 and G-H) ph-STAT5. * = One or more bands associated with ph/total quantification was not assessed or was imperfect. Blots were cut at indicated locations prior to primary antibody incubation.

Figure S4

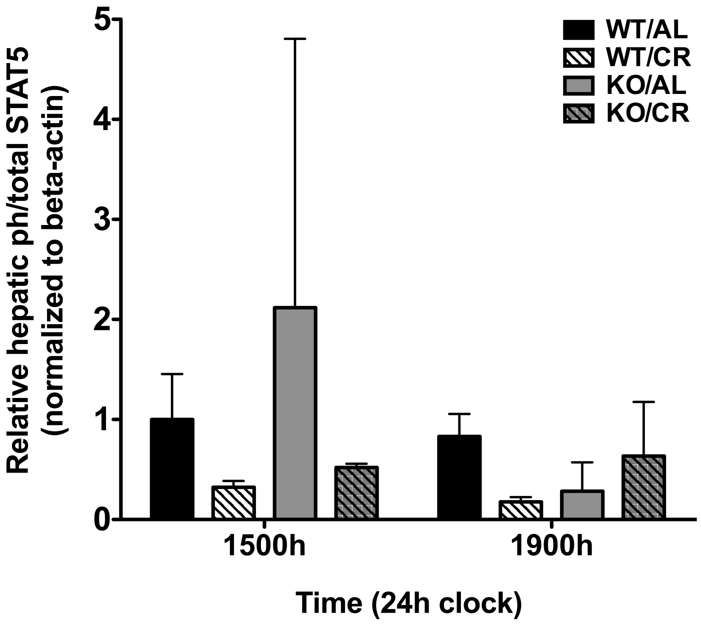

Figure S4. Ph-total hepatic STAT5 levels in AL-fed and CR-fed WT and FGF21-KO mice. n = 4-5 per genotype per diet per time point. Data normalized to WT/AL mice at 1500h. All between-group analyses were performed using a two-way ANOVA with a Bonferroni *post hoc* test at each time point.

Figure S5

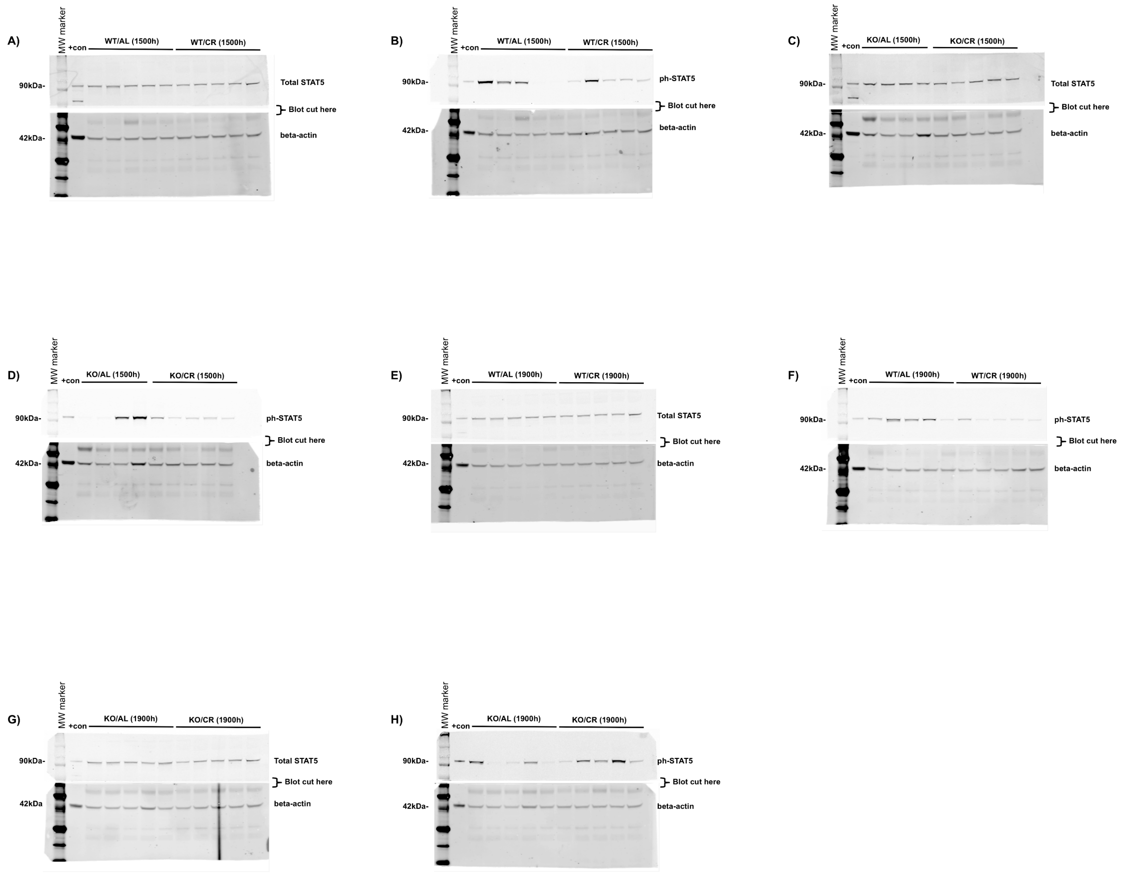

Figure S5. Blots used to quantify hepatic ph-total STAT5 levels in AL-fed and CR-fed WT and FGF21-KO mice. Hepatic protein levels of A) Total-STAT5 in WT/AL and WT/CR mice at 1500h, B) ph-STAT5 in WT/AL and WT/CR mice at 1500h, C) Total-STAT5 in KO/AL and KO/CR mice at 1500h, D) ph-STAT5 in KO/AL and KO/CR mice at 1500h, E) Total-STAT5 in WT/AL and WT/CR mice at 1900h, F) ph-STAT5 in WT/AL and WT/CR mice at 1900h, G) Total-STAT5 in KO/AL and KO/CR mice at 1900h and H) ph-STAT5 in KO/AL and KO/CR mice at 1900h. + con = extract from UT-7 cells treated with GM-CSF (positive control for STAT5 phosphorylation). Blots were cut at indicated locations prior to primary antibody incubation.
